# Evaluation of daily time spent in transportation and traffic-influenced microenvironments by urban Canadians

**DOI:** 10.1007/s11869-017-0532-6

**Published:** 2017-11-30

**Authors:** Carlyn J. Matz, David M. Stieb, Marika Egyed, Orly Brion, Markey Johnson

**Affiliations:** 1 Air Health Effects Assessment Division, Health Canada, 269 Laurier Ave W, PL 4903C, Ottawa, ON K1A 0K9 Canada; 2 Population Studies Division, Health Canada, 445-757 West Hasting St., Federal Tower, Vancouver, BC V6C 1A1 Canada; 3 Population Studies Division, Health Canada, 101 Tunney’s Pasture Dr., PL 0201A, Ottawa, ON K1A 0K9 Canada; 4 Air Health Science Division, Health Canada, 269 Laurier Ave W, PL 4903C, Ottawa, ON K1A 0K9 Canada

**Keywords:** Time-activity patterns, Traffic, Transportation, Nitrogen dioxide, Survey, Canada

## Abstract

Exposure to traffic and traffic-related air pollution is associated with a wide array of health effects. Time spent in a vehicle, in active transportation, along roadsides, and in close proximity to traffic can substantially contribute to daily exposure to air pollutants. For this study, we evaluated daily time spent in transportation and traffic-influenced microenvironments by urban Canadians using the Canadian Human Activity Pattern Survey (CHAPS) 2 results. Approximately 4–7% of daily time was spent in on- or near-road locations, mainly associated with being in a vehicle and smaller contributions from active transportation. Indoor microenvironments can be impacted by traffic emissions, especially when located near major roadways. Over 60% of the target population reported living within one block of a roadway with moderate to heavy traffic, which was variable with income level and city, and confirmed based on elevated NO_2_ exposure estimated using land use regression. Furthermore, over 55% of the target population ≤ 18 years reported attending a school or daycare in close proximity to moderate to heavy traffic, and little variation was observed based on income or city. The results underline the importance of traffic emissions as a major source of exposure in Canadian urban centers, given the time spent in traffic-influenced microenvironments.

**Electronic supplementary material:**

The online version of this article (10.1007/s11869-017-0532-6) contains supplementary material, which is available to authorized users.

## Introduction

Traffic-related air pollution (TRAP) is associated with adverse cardiorespiratory health effects, including exacerbation of asthma, incident asthma, reduced lung function, myocardial infarction, progression of atherosclerosis, and cardiovascular mortality (Health Effects Institute 2010). Recent evidence also links TRAP exposure to a wide array of other adverse health impacts throughout the life course ranging from adverse birth outcomes to dementia (Stieb et al. 2016; Oudin et al. 2016; Pedersen et al., 2013; Chen et al. 2017a). TRAP is a complex mixture consisting of particle and gaseous components and includes both primary air pollutants, which are directly emitted from vehicle exhaust, brake, and tire wear, as well as secondary air pollutants that form from reactions of primary pollutants in the atmosphere. Pollutants commonly measured as surrogates for traffic exposure include carbon monoxide (CO), nitrogen dioxide (NO_2_), elemental or black carbon (EC or BC), benzene, and ultrafine particles (UFP). Traffic-related emissions impact ambient air quality and can be a major source of exposure to air pollutants in urban areas. In Canada, it has been estimated that about ten million people (approximately 32% of the population) live within 500 m of highways or 100 m of major urban roads (Brauer et al. 2013). In urban areas, traffic is also the principal source of exposure to noise (Allen et al. 2009; Davies et al. 2009), which has been associated with a variety of adverse impacts from annoyance to ischemic heart disease (World Health Organization 1999, 2011).

For many epidemiological studies, traditional exposure assessment methods have relied on use of central site monitors or predicted ambient concentrations at residence to assign exposures to the study population. However, these approaches can lead to exposure misclassification and/or bias by not incorporating a person’s daily movements and activities into the exposure estimate (Setton et al. 2011; Baxter et al. 2013; Ozkaynak et al. 2013). Exposure estimates have been improved when considering daily time spent away from the home and daily time in transit (Dons et al. 2011; de Nazelle et al. 2013; Dias and Tchepel 2014; Ragettli et al. 2015; Shekarrizfard et al. 2016). By characterizing both time spent and pollutant concentrations in individual microenvironments, it is possible to identify locations that contribute substantially to overall exposure and to guide exposure mitigation strategies (Almeida-Silva et al. 2014; Tchepel et al. 2014; Williams and Knibbs 2016).

The Canadian Human Activity Pattern Survey (CHAPS) 2 was conducted by Health Canada to provide information on daily time-activity patterns, potential exposure to environmental contaminants, occupational activities, and housing characteristics (Matz et al. 2014). The CHAPS 2 results can be used to inform exposure assessment, risk assessment, and risk management activities related to environmental health. In the CHAPS 2 survey, several questions were focussed on daily time spent in activities and locations associated with TRAP exposure. Time spent on or near roadways, especially roadways with heavy traffic, can be a substantial contributor to daily exposure to TRAP and to air pollution in general. Roadways and transit locations (e.g., locations associated with commuting activities) represent exposure hot spots, with pollutant concentrations exceeding those measured at regional outdoor monitors (Weichenthal et al. 2015).

Initial findings from CHAPS 2 indicated that age was a significant predictor of daily time spent in a vehicle, with adults spending the most time (1.5 h per day) in this microenvironment (Matz et al. 2014). In the present study, our objectives were to (1) generate population-representative estimates of daily time spent in transportation and traffic-influenced microenvironments, as possible sources of TRAP exposure for urban Canadians; (2) identify factors influencing these time-activity patterns; (3) evaluate internal consistency between 24-h recall diary and survey responses pertaining to time spent in traffic-influenced microenvironments; and (4) confirm self-reported residential proximity to roadways with moderate to heavy traffic based on estimated ambient NO_2_ concentration at the respondent’s residence from a land-use regression (LUR) model.

## Methods

### Canadian Human Activity Pattern Survey 2 survey data

A detailed description of survey methodology and study population for CHAPS 2 has previously been published (Matz et al. 2014). Briefly, a random digit dialing survey was conducted, in 2010–2011, using computer-assisted telephone interview (CATI) technology to collect time-activity data and questionnaire responses. The target population was Canadian residents of all ages with a telephone residing in five urban areas (Vancouver, Edmonton, Toronto, Montreal, and Halifax) and two rural regions (Haldimand-Norfolk, Ontario, and Annapolis Valley-Kings County, Nova Scotia). For the present analysis, only data from urban respondents were considered as traffic-related air pollution is principally associated with urban centers. The CHAPS 2 survey instrument was based on the original CHAPS survey (Leech et al. 1996) and consisted of three main components: questions regarding respondent characteristics and household composition, a 24-h recall diary, and a supplemental questionnaire covering activities related to exposures to specific contaminants, dwelling characteristics, socio-economic status (SES), and health status. The 24-h recall diary was used to collect time-activity information as respondents described their activities starting from midnight of the previous day. The complete survey instrument is available in Supplementary Materials of the methodology publication (Matz et al. 2014). This study was approved by Health Canada’s Research Ethics Board.

Sampling was conducted in summer 2010 and winter 2011. A total of 3551 urban respondents participated in the survey. Details of the response rates and representativeness of the survey are presented in Matz et al. (2014). Survey weights were calculated to account for oversampling of certain age groups, adjustments for non-response, and to allow for generalization of survey results to the entire target area population.

The main purpose of this analysis was to evaluate daily time spent in transportation and traffic-influenced microenvironments. Analysis of time spent in vehicles, engaged in active transportation, or otherwise on or near a roadway was based on data collected in the 24-h recall diary. Time spent in parking lots or gas stations was based on data collected in the questionnaire. Information on time spent in moderate to heavy traffic was collected in both the 24-h recall diary and supplemental questionnaire. For the recall diary of the previous day, any time a respondent indicated that he/she was in a vehicle (e.g., car, truck), in transit (e.g. bus, rapid transit), engaged in active transport (e.g., walking, running, cycling), or waiting at a transit stop, the respondent was asked if this activity was conducted on or near a roadway with moderate to heavy traffic, and if so, for how long. For the supplemental questionnaire, respondents were asked whether in the previous day they had spent time in a car, van, truck, or bus, in moderate to heavy traffic or running, walking, or standing along a road with moderate to heavy traffic, and if so, for how long. These data were collected in both the diary and supplemental questionnaire as respondents may not remember to report all activities during the recall process (Klepeis et al. 2001). In the present study, the 24-h recall diary was considered the primary data source and the supplemental questionnaire questions on traffic-related exposures were used to assess internal consistency. For the recall diary and supplemental questionnaire, a roadway with moderate to heavy traffic was defined as one that has a substantial amount of traffic for several hours of the day, such as a main thoroughfare, a busy boulevard, or a highway. Survey details, including specific questions evaluated in this publication, are provided in the Supplemental Materials.

### Statistical analysis

CHAPS 2 has a complex design, involving stratification by location and season and clustering by households. The reported statistical analyses accounted for this structure by using the respective parameters in all software procedures (Heeringa et al. 2010). Sampling weights were used to make the estimates generalizable to the CHAPS 2 target population. The sampling errors were estimated using Taylor linearization method. Summary statistics (means, percentiles, and percentages) were estimated using SAS SURVEYMEANS and SURVEYFREQ procedures and SUDAAN DESCRIPT procedure. Regression analysis was performed using SAS SURVEYREG procedure. Odds ratios were estimated by fitting logistic models using SAS SURVEYLOGISTIC procedure. Significance of the model parameters was tested using Wald chi-square test statistics. Differences in means were tested using Wald *F* statistic. Significance level used in the analysis was 0.05. The analysis was performed with SAS Enterprise Guide 4.2 (SAS Institute Inc., Cary, NC) and SUDAAN 10.0.1. The estimated sampling variability was evaluated using Statistics Canada’s guidelines for reliability of household survey data (Statistics Canada 2014): estimates with high sampling variability (coefficient of variation > 16.5 and ≤ 33.3%) were interpreted with caution, and estimates with very high variability (coefficient of variation > 33.3%) were suppressed.

For both the 24-h recall diary and supplemental questionnaire, time spent in each microenvironment was estimated for the entire target population (i.e., including both those who did and did not spend time in the microenvironment) and restricted to “doers” (i.e., only those who spent at least 1 min of time in the microenvironment or activity). The doers provide specific information about that portion of the total population that was in a given microenvironment. Where sample size was sufficient (> 10 respondents), analysis was also conducted for four age groups, 0–4 years, 5–18 years, 19–64 years, and 65+ years. As age is a predictor of time-activity patterns (Matz et al. 2014), it was anticipated the daily activity patterns would be similar within these age groups.

To evaluate internal consistency of the survey responses, Spearman correlation analysis and *t* tests were used to evaluate the relationship between time spent in traffic reported in the 24-h recall diary and supplemental questionnaire.

Income, age, education level, employment status, and city of residence were chosen a priori as covariates for analysis. Income was evaluated as above or below low-income cut-offs (LICO), defined by Statistics Canada as thresholds below which a household will likely devote a larger share of its income to food, shelter, and clothing compared to an average family, adjusted for household size and community size (Statistics Canada 2013).

### NO_2_ exposure

In order to provide external confirmation of self-reported residential proximity to moderate to heavy traffic, NO_2_ concentrations at residence were examined. Residential estimates of ambient NO_2_ concentrations were derived from a national LUR model combined with deterministic gradients to capture regional and local scale variation. The development of the national LUR model has been described in previous publications (Hystad et al. 2011; Crouse et al. 2015). Briefly, a national LUR model was developed to predict regional NO_2_ concentrations using National Air Pollution Surveillance (NAPS; http://www.ec.gc.ca/rnspa-naps/) monitoring data collected in 2006 (Hystad et al. 2011; Crouse et al. 2015). Final LUR model predictors include road length within 10 km, 2005–2011 satellite NO_2_ estimates, area of industrial land use within 2 km, and summer rainfall. The model explains 73% of the variation in mean annual NAPS concentrations from 2006, with a root mean square error of 2.9 ppb. Local scale variation due to vehicle emissions was modeled using deterministic gradients from the literature and kernel density measures as described by Crouse et al. (2015).

NO_2_ concentrations were estimated for each postal code based on representative points. Representative points are unique coordinates within each postal code that reflect the postal code centroid for postal codes capturing a single block, or multiple central points along a line for postal codes larger than one block (Statistics Canada 2016). In urban areas, postal codes typically represent a single city block or a single apartment building. For postal codes with more than one representative point (e.g., postal codes larger than one city block), NO_2_ estimates for all representative points within the postal code were averaged to generate the postal code level estimate. Postal code level NO_2_ concentrations were assigned to CHAPS 2 respondents based on self-reported residential postal code. Mean NO_2_ concentrations were estimated for subpopulations defined by living within one block of a roadway with moderate to heavy traffic, income level, or education. Differences between subpopulation mean NO_2_ concentrations were tested using *t* test. The analysis was performed using DESCRIPT procedure in SUDAAN 10.0.1.

## Results

### Microenvironments influenced by traffic-related air pollution

Time spent in microenvironments which are influenced by TRAP is summarized in Table 1. Among those who spent any time in these microenvironments (“doers”), over 1 h a day on average was spent in a car or on a bus and over 30 min a day on average was spent walking. The time in these microenvironments increases to approximately 3.5, 2.9, and 2.0 h, respectively, when considering the 95th percentile of the doers, representing a subgroup with potentially high TRAP exposure. Overall, 16.7 and 60.2% of the target population spent time in an enclosed or underground parking garage (20.4 min on average) or surface parking (10.0 min on average), respectively. Only 12.5% of the target population went to a gas station and of these, 57.4% pumped fuel and 30.8% were in a vehicle while someone else pumped fuel. A summary of time spent by doers in all microenvironments that may be influenced by vehicle emissions, collected in the recall diary, is provided in Supplemental Material Table S1.

**Table 1:** Mean daily time spent in traffic-influenced microenvironments

Microenvironment	Survey group	Weighted % of target population (*N*)	Mean daily time (95% CI) (min)	95th percentile (min)
Car^a^	All respondents	100 (3551)	46.1 (42.0–50.1)	164.5
	Doers^c^	59.5 (2116)	77.3 (71.6–83.0)	208.1
Bus^a^	All respondents	100 (3551)	8.1 (6.0–10.3)	49.4
	Doers	12.2 (347)	66.3 (51.1–81.5)	173.8
Walking^a^	All respondents	100 (3551)	13.5 (11.9–15.1)	69.5
	Doers	37.2 (1167)	36.2 (32.7–39.8)	119.3
Parking garage^b^	All respondents	100 (3515)	3.4^d^ (1.5–5.3)	9.0
	Doers	16.7 (618)	20.4^d^ (9.8–31.5)	Data suppressed^e^
Parking lot^b^	All respondents	100 (3511)	6.0 (5.2–6.9)	20.4
	Doers	60.2 (2118)	10.0 (8.7–11.3)	28.8
Gas station^b^	All respondents	100 (3513)	1.6 (1.2–1.9)	9.0
	Doers	12.5 (399)	12.4 (9.9–14.9)	26.5

^a^Based on 24-h recall diary

^b^Based on supplemental questionnaire

^c^Those who reported spending time in microenvironment

^d^High sampling variability based on Statistics Canada guidelines (Statistics Canada 2014), interpret with caution

^e^Due to very high sampling variability based on Statistics Canada guidelines (Statistics Canada 2014), data are suppressed

### Time spent in transportation and traffic

#### 24-h recall diary

Based on the 24-h recall diary, more people reported, in general, spending time in a vehicle (Table 2) than using active transportation (Table 3) for each age group. Specifically, it was estimated that ≥ 62.3% of the target population, of each age group, reported being in a vehicle on road, while ≤ 41.5% of each age group spent time in active transportation. Additionally, mean daily time in a vehicle was typically greater than time spent in active transportation. It was also estimated that at least 43.6% of the target population for each age group reported being in moderate to heavy traffic while in an on-road location, for an average of 39.3–54.2 min for the four age groups. In comparison, ≤ 21.7% of each age group was in moderate to heavy traffic while engaged in active transportation, for an average of 20.7–38.9 min. For all respondents, mean total daily time in on- or near-road microenvironments (Table 4) ranged from 56.3–101.0 min, with 22.0–41.0 min on average in moderate to heavy traffic. In the doer group, the mean daily time on- or near-road was 72.3–111.4 min, with 28.1–45.2 min in moderate to heavy traffic, and for those who spent any time in the vicinity of moderate to heavy traffic, mean daily time in moderate to heavy traffic ranged from 42.8–58.2 min. Age was a significant predictor for daily time spent in a vehicle on road and associated time in moderate to heavy traffic (*p* < 0.001), as well as total daily time spent on-or near road and associated time in moderate to heavy traffic (*p* < 0.001). Age was not a significant predictor for daily time spent in active transportation (*p* = 0.25), but was a significant predictor for the associated time in moderate to heavy traffic (*p* = 0.0063). No summer-winter or weekday-weekend differences were noted for time spent in active transportation.

**Table 2:** Recall diary: mean daily time spent in a vehicle on-road and in moderate to heavy traffic while in a vehicle on-road

Age group	Survey group	Weighted % of target population (*N*)	Mean daily time (95% CI) (min)	
			On road in a vehicle	On road in moderate to heavy traffic
0–4 years	All respondents	100 (338)	41.7 (28.8–54.6)	17.2^c^ (10.3–24.0)
	Doers^a^	62.3 (217)	67.0 (49.2–84.8)	27.6^c^ (17.8–37.3)
	Traffic^b^	43.6 (164)	76.2 (53.2–99.3)	39.3 (27.2–51.4)
5–18 years	All respondents	100 (527)	45.5 (38.7–52.2)	20.8 (15.9–25.8)
	Doers	73.3 (387)	62.0 (53.4–70.7)	28.4 (21.9–35.0)
	Traffic	48.1(278)	73.5 (63.4–83.6)	43.3 (35.2–51.4)
19–64 years	All respondents	100 (1960)	77.2 (65.4–89.1)	32.9 (30.0–35.8)
	Doers	78.4 (1535)	98.5 (84.2–112.8)	42.0 (38.5–45.4)
	Traffic	60.7 (1258)	100.7 (83.7–117.7)	54.2 (50.5–57.9)
65+ years	All respondents	100 (726)	51.8 (44.3–59.4)	23.8 (20.0–27.6)
	Doers	65.7 (489)	78.9 (69.0–88.7)	36.2 (31.0–41.5)
	Traffic	49.9 (395)	84.6 (73.0–96.2)	47.7 (41.9–53.5)

^a^Those who reported spending time in a vehicle on-road

^b^Those who reported spending any time in moderate to heavy traffic while in a vehicle on-road

^c^High sampling variability, based on Statistics Canada guidelines (Statistics Canada 2014), interpret with caution

**Table 3:** Recall diary: mean daily time spent in active transportation and in moderate to heavy traffic while in active transportation

Age group	Survey group	Weighted % of target population (*N*)	Mean daily time (95% CI) (min)	
			Active transportation	Active transportation in moderate to heavy traffic
0–4 years	All respondents	100 (338)	10.1^c^ (4.9–15.2)	Data suppressed^d^
	Doers^a^	28.2 (62)	35.6^c^ (20.8–50.4)	Data suppressed^d^
	Traffic^b^	11.3 (26)	62.8^c^ (37.1–88.5)	38.9^c^ (14.4–63.3)
5–18 years	All respondents	100 (527)	16.1 (11.2–21.0)	3.1^c^ (1.8–4.3)
	Doers	41.5 (210)	38.8 (28.9–48.7)	7.4^c^ (4.6–10.3)
	Traffic	14.9 (78)	43.4^c^ (28.3–58.5)	20.7 (15.0–26.4)
19–64 years	All respondents	100 (1960)	15.6 (13.4–17.7)	6.4 (4.9–7.9)
	Doers	41.2 (718)	37.7 (33.4–42.1)	15.5 (12.3–18.8)
	Traffic	21.7 (384)	46.2 (40.5–52.0)	29.5 (24.7–34.2)
65+ years	All respondents	100 (726)	14.3 (10.8–17.9)	3.7 (2.6–4.8)
	Doers	30.9 (224)	46.4 (37.4–55.4)	11.9 (8.7–15.1)
	Traffic	14.8 (108)	52.8 (39.8–65.9)	24.8 (20.1–29.5)

^a^Those who reported spending time in active transportation

^b^Those who reported spending any time in moderate to heavy traffic while in active transportation

^c^High sampling variability based on Statistics Canada guidelines (Statistics Canada 2014), interpret with caution

^d^Due to very high sampling variability based on Statistics Canada guidelines (Statistics Canada 2014), data are suppressed

**Table 4:** Recall diary: mean daily time spent on- or near-road and in moderate to heavy traffic

Age group	Survey group	Weighted % of target population (*N*)	Mean daily time (95% CI) (min)	
			On or near road	On or near road in moderate to heavy traffic
0–4 years	All respondents	100 (338)	56.3 (43.2–69.3)	22.0^c^ (14.5–29.6)
	Doers^a^	77.3 (245)	72.8 (57.3–88.4)	28.5 (22.1–34.1)
	Traffic^b^	51.5 (178)	85.7 (65.4–106.1)	42.8 (31.1–54.5)
5–18 years	All respondents	100 (527)	64.0 (55.5–72.6)	24.9 (19.5–30.3)
	Doers	88.6 (457)	72.3 (62.8–81.7)	28.1 (22.1–34.1)
	Traffic	54.6 (309)	86.0 (74.7–97.4)	45.6 (37.8–53.4)
19–64 years	All respondents	100 (1960)	101.0 (88.9–113.1)	41.0 (37.6–44.4)
	Doers	90.6 (1724)	111.4 (98.4–124.5)	45.2 (41.6–48.9)
	Traffic	70.5 (1407)	115.5 (100.0–130.9)	58.2 (54.2–62.1)
65+ years	All respondents	100 (726)	71.6 (63.0–80.2)	28.4 (24.3–32.5)
	Doers	75.4 (565)	95.0 (85.2–104.7)	37.6 (32.6–42.6)
	Traffic	56.4 (447)	101.5 (90.6–112.4)	50.3 (44.7–55.9)

^a^Those who reported spending time in on- or near-road locations

^b^Those who reported spending any time in moderate to heavy traffic

^c^High sampling variability based on Statistics Canada guidelines (Statistics Canada 2014), interpret with caution

#### Supplemental questionnaire

Daily time spent in the vicinity of moderate to heavy traffic, in various microenvironments, was also assessed in the supplemental questionnaire to assess internal consistency of the CHAPS 2 survey. The estimated portions of the target population and time spent in the moderate to heavy traffic while in a vehicle, while running, walking, or standing along a roadside, and total time are available in the Supplemental Materials (Tables S2, S3, and S4, respectively). From the data collected in the supplemental questionnaire, age was a significant predictor for time spent in moderate to heavy traffic while in a vehicle (*p* = 0.002) and total time spent in traffic-related microenvironments (*p* = 0.0014). In comparison, age was not a significant predictor for time spent in moderate to heavy traffic while running, walking, or standing along a roadside (*p* = 0.27).

#### Internal consistency

Measures of time spent in moderate to heavy traffic while being in a vehicle and of total time in moderate to heavy traffic were highly correlated (*ρ* = 0.75–0.83) between the 24-h recall diary and the supplemental questionnaire, for all respondents and by age group. Similar degrees of correlation were also observed by household income level, employment status, and education level (*ρ* ≥ 0.71) (Supplemental Material Table S5). Although highly correlated, respondents reported spending more time in moderate to heavy traffic in the supplemental questionnaire compared to the 24-h recall diary; on average, a difference of 9.1 min more was reported while in a vehicle (*p* < 0.05) and 15.0 min more overall (*p* < 0.05). Mean differences for reported times in moderate to heavy traffic between the 24-h recall diary and supplemental questionnaire were variable by age group, household income level, employment status, and education level (Supplemental Material Table S6). In some instances, wide 95% confidence intervals were estimated, reflecting the smaller sample sizes in the subgroups being compared.

### Proximity to roadway

Overall, an estimated 60.6% (95% CI 57.9–63.3%) of the target population reported living within one block of a roadway with moderate to heavy traffic, and this was reported more commonly by those with household income ≤ LICO, but not by those with less than university education (Table 5). It was also reported more commonly in Toronto, Halifax, Vancouver, and Edmonton than in Montreal. Additionally, an estimated 55.7% (95% CI 50.3–61.1%) of the target population up to 18 years reported attending a school or daycare within one block of a roadway with moderate to heavy traffic. This was not significantly associated with income, but the odds were significantly greater for Toronto compared to the reference city, Montreal (Table 6). Estimates based on LUR modeling indicated that ambient NO_2_ concentrations were greater at the residences of those who reported living within one block of a roadway with moderate to heavy traffic compared to those that did not (19.2 ppb vs. 16.7 ppb, *p* < 0.001), providing a confirmation of the supplemental questionnaire responses. A significantly greater NO_2_ exposure was also noted for those with household income ≤ LICO (19.6 ppb vs. 17.7 ppb, *p* = 0.002). With respect to differences by education level, NO_2_ exposure was significantly lower for those who had completed secondary education compared to those that had completed university (18.0 ppb vs. 19.0 ppb, *p* = 0.027).

**Table 5:** Prevalence of residing within one block of a roadway with moderate to heavy traffic and association with income, education, and city

Subgroup	Weighted % residing within one block of roadway with moderate to heavy traffic (*N*)	OR (95% confidence interval)	
Target population	60.6 (2150)		
Income ≤ LICO	72.0 (413)	1.828 (1.326–2.520)*	
Income > LICO	58.5 (1274)	1.0	
Less than secondary education^a^	63.8 (154)	1.114 (0.678–1.831)	
Completed secondary education	64.1 (865)	1.127 (0.846–1.500)	
Completed university	61.3 (666)	1.0	
		Adjusted for income	Adjusted for education
Vancouver	60.1 (447)	1.733 (1.226–2.450)*	1.617 (1.126–2.321)*
Edmonton	56.4 (437)	1.514 (1.083–2.116)*	1.351 (0.950–1.921)
Toronto	71.1 (484)	2.866 (2.047–4.012)*	2.677 (1.890–3.791)*
Halifax	67.2 (437)	2.389 (1.650–3.459)*	2.262 (1.558–3.285)*
Montreal^b^	47.6 (345)	1.0	1.0

*Statistically significant at *p* < 0.05

^a^Educational attainment was only ascertained for respondents ≥ 18 years

^b^Montreal is the reference city as it has the lowest estimated percentage of population residing within one block of a roadway with moderate to heavy traffic

**Table 6:** Prevalence of attending daycare or school within one block of a roadway with moderate to heavy traffic and association with income and city

Variable	Weighted % attending daycare or school within one block of roadway with moderate to heavy traffic (*N*)	OR (95% confidence interval)
Target population ≤ 18 years	55.7 (411)	
Income ≤ LICO	52.7 (64)	0.799 (0.421–1.518)
Income > LICO	58.6 (282)	1.0
		Adjusted for income
Vancouver	51.2 (85)	1.003 (0.532–1.891)
Edmonton	51.8 (90)	1.396 (0.758–2.571)
Toronto	62.9 (97)	2.015 (1.053–3.858)*
Halifax	60.4 (67)	1.555 (0.782–3.093)
Montreal^a^	48.5 (72)	1.0

*Statistically significant at *p* < 0.05

^a^Montreal is the reference city as it has the lowest estimated percentage of population attending a daycare or school within one block of a roadway with moderate to heavy traffic

Those who reported living within one block of a roadway with moderate to heavy traffic spent more time in the vicinity of moderate to heavy traffic compared to those that did not report living in close proximity to traffic (Table 7). From the diary, daily time spent in moderate to heavy traffic while in active transportation was greater among those living in proximity to traffic for those 0–4 years (Δ = 20.4 min, *p* = 0.0427), 19–64 years (Δ = 9.5 min, *p* = 0.0015), and 65+ years (Δ = 8.7 min, *p* = 0.0043), though total time in active transportation was not different between the groups. From the supplemental questionnaire, time spent in moderate to heavy traffic while walking, running, or standing along a roadside was greater for those 0–4 years (Δ = 19.9 min, *p* = 0.0159) and 19–64 years (Δ = 8.7 min, *p* < 0.0001). No differences were noted for time spent in moderate to heavy traffic associated with being in a vehicle or total time in moderate to heavy traffic between those who reported living within one block of a major roadway and those who did not.

**Table 7:** Mean difference in daily time: those who reported living within one block of a roadway with moderate to heavy traffic vs. those who did not

Age group (sample size living near roadway, sample size that do not)	Time in active transportation^a^ (min)		Time in moderate to heavy traffic associated with active transportation^a^ (min)		Time in moderate to heavy traffic while in a car, van, truck, or bus^b^ (min)		Time in moderate to heavy traffic while running, walking, or standing along a roadside^b^ (min)		Time in moderate to heavy traffic while in car, van, truck, or bus or running, walking, or standing along a roadside^b^ (min)	
	Difference	95% CI	Difference	95% CI	Difference	95% CI	Difference	95% CI	Difference	95% CI
0–4 years (164, 166)	15.9^c^	− 8.2 – 40.0	20.4^c,^*	0.7 – 40.2	− 6.9	− 19.9 – 6.1	19.9*	3.8 – 36.0	12.9	− 7.8 – 33.6
5–18 years (1216, 714)	− 1.6	− 20.9 – 17.8	5.0	− 0.8 – 10.8	5.1	− 7.3 – 17.4	3.6	− 2.5 – 9.8	8.6	− 7.1 – 24.2
19–64 years (280, 234)	1.9	− 7.2 – 11.0	9.5*	3.6 – 15.3	5.1	− 13.9 – 24.1	8.7*	4.7 – 12.7	13.6	− 7.1 – 34.3
65+ years (490, 225)	− 1.5	− 26.8 – 23.7	8.7*	2.8 – 14.7	− 1.1	− 10.4 – 8.3	4.7	− 2.5 – 11.9	3.2	− 8.7 – 15.2

*Significant values represented in bold

^a^Based on 24-h recall diary

^b^Based on supplemental questionnaire

^c^Interpret with caution, due to small sample size

## Discussion

The main purpose of this study was to evaluate daily time spent by urban Canadians in transportation and traffic-influenced microenvironments. The CHAPS 2 survey results indicated that urban Canadians spend approximately 4–7% of daily time (56.3–101.0 min) in on- or near-road locations by age group, and this increased to 5–8% (72.8–111.4 min) among individuals who spent any time in those locations. Children (0–18 years), considered a sensitive subpopulation to the adverse effects of air pollution, spend on average approximately 1 h a day on or near roadways. These results are consistent with other recent studies, which have reported about 6% of daily time spent in transportation microenvironments (Dons et al. 2011, 2012; de Nazelle et al. 2013). Dons et al. (2011) further identified that for full-time workers, daily time spent in transportation increases to 7.8% compared to 6.2% for the entire study population. Additionally, motorized vehicles were the most commonly used mode of transportation and represented larger portions of daily time compared to active transportation (Dons et al. 2011; de Nazelle et al. 2013).

Results from the present study highlight the important role of traffic as a major source of air pollution exposure for Canadians living in urban centers. For each of the age groups considered in this study, a substantial amount of daily time was spent in transportation-influenced microenvironments with moderate to heavy traffic. Importantly, a significant proportion of the target population engaged in active transportation near roadways (approximately 30–40% across the age groups) and approximately 11–22% of the target population did so in the proximity of moderate to heavy traffic. For some age groups, living within one block of a roadway with moderate to heavy traffic was associated with increased daily time spent in traffic while engaged in active transportation. This implies that there may be age-related differences in the degree of exposure misclassification when exposure is assigned based on place of residence (Setton et al. 2011; Gurram et al. 2015; Ragettli et al. 2015). Overall, the results highlight the potential impact of traffic emissions on the population and that access to active transportation options in areas or along routes less impacted by vehicle emissions may have public health benefits.

Compared to time spent at home, time spent in transportation locations accounts for a small portion of the day but has been demonstrated to account for a greater portion of total daily exposure to air pollution (Dons et al. 2011, 2012; de Nazelle et al. 2013; Dias and Tchepel et al. 2014; Lane et al. 2015). In each of these studies, the percent contribution of transportation microenvironments to time-weighted exposure for black carbon, PM_2.5_, ultrafine particles, or NO_2_ was ~ 2–4 times greater than the percent of daily time associated with transportation, since transportation locations typically have greater concentrations of these pollutants compared to home and work locations. Additionally, greater exposure error and bias in risk estimates have been reported when study subjects spend more time away from home, travel greater distances, or spend more time in travel, compared to estimates based on residence location only (Setton et al. 2011; Gurram et al. 2015; Ragettli et al. 2015). People that commute by car are exposed to higher levels of air pollutants than active commuters, including both pedestrians and cyclists (Cepeda et al. 2017). However, modes of active transportation can result in greater personal exposure per trip compared to traveling in vehicle for a given route, due to the increased travel time in areas with higher levels of traffic (Good et al. 2016), as well as a larger inhaled dose due to the increased breathing rate (Dons et al. 2012; Ragettli et al. 2015; Cepeda et al. 2017). Nonetheless, there is evidence that outdoor physical activity results in a net health benefit, despite the increased exposure to air pollutants (Andersen et al. 2015, Cepeda et al. 2017).

Indoor air quality at home, work, or school, can also be influenced by traffic emissions, especially when these indoor locations are situated near a major roadway. Given that Canadians spend approximately 21 h per day indoors (Matz et al. 2014), this represents an additional and potentially significant contribution of traffic emissions to daily exposure to air pollution. It has been estimated that approximately 32% of the Canadian population lives within 100 m of a major urban road or 500 m of a highway (Brauer et al. 2013). In the present study, 60.6% of the target population reported living within one block of a roadway with moderate to heavy traffic. This difference may be explained, at least in part, by differences in the population under consideration: in the present analysis, the sample was restricted to the urban population of five major Canadian cities, in contrast to the Brauer et al. (2013) report which included both urban and rural populations. In addition, 55.7% of the target population up to 18 years, in the present study, reported attending a school or daycare within one block of a roadway with moderate to heavy traffic. In a study of ten major Canadian cities, 16.3 and 36.1% of public elementary schools, based on geocoding, were within 75 and 200 m of a major roadway, respectively (Amram et al. 2011). For Montreal and Vancouver, two of the cities included in CHAPS 2, over 50% of the public elementary schools were within 200 m of a major roadway. Moreover, schools in neighborhoods with higher dwelling density and lower median income were closer to a major roadway. In the present study, household income did not have a significant association with attending a school or daycare within one block of a roadway with moderate to heavy traffic, and little variation was observed between cities. Concerns over potential health effects in children and youth have led to recent investigations of possible interventions to reduce exposure to TRAP in schools (MacNeill et al. 2016, van der Zee et al. 2017).

Living within one block of a roadway with moderate to heavy traffic and elevated NO_2_ exposure (an indicator of traffic exposure) were both associated with lower household income in this study. Similar results have been reported in previous Canadian evaluations. Lower SES residential areas were more prevalent within 200 m of a major highway, compared to high SES areas, in Vancouver, Toronto, and Montreal (Canadian Institute for Health Information 2011). An opposite trend was noted for Edmonton, which was attributed to the major highway passing through suburban areas and not the city center. Additionally, several studies have evaluated NO_2_ levels and social geography in major Canadian cities, identifying predictors of potential susceptibility or environmental injustice. Higher NO_2_ levels have been associated with various neighborhood SES characteristics in Toronto, Montreal, and/or Vancouver, including higher unemployment (Crouse et al. 2009), lower household income (Buzzelli and Jerrett 2007; Crouse et al. 2009; Su et al. 2010; Pinault et al. 2016a, b), people living alone (Crouse et al. 2009; Pinault et al. 2016a), low education (Buzzelli and Jerrett 2007), single-parent homes (Buzzelli and Jerrett 2007; Pinault et al. 2016b), visible minorities (Crouse et al. 2009; Pinault et al. 2016b), and linguistic isolation (Pinault et al. 2016a). However, for both Toronto (Buzzelli and Jerrett 2007) and Montreal (Crouse et al. 2009), elevated levels of NO_2_ were also associated with indicators of higher SES, including greater dwelling value, high-status occupation, higher education, and/or higher income, in some city center neighborhoods. These results were attributed to gentrification of downtown areas in Toronto and, for Montreal, historically affluent enclaves and presence of universities in the city center. Although these results demonstrate that the relationship between measures of SES and TRAP exposure is not entirely straightforward and may differ within and between cities, there is growing evidence that lower SES residential areas in Canada may be more impacted by the negative effects of traffic.

### Strengths and limitations

A key strength of CHAPS 2 is the large sample size, including over 3500 respondents from five major urban centers, which provided detailed time-activity data that can be evaluated by age group. Twenty-four-hour recall diaries are a standard method for collecting time-activity information (Leech et al. 1996; Klepeis et al. 2001) and the reproducibility of the data has been established (Freeman et al. 1999; Wu et al. 2011). Use of global positioning system (GPS) devices has been proposed to improve the accuracy of location data and reduce participant burden (Breen et al. 2014; Nethery et al. 2014); however, these methods are not feasible for studies with a large number of participants.

In this study, internal consistency (high correlation) was observed between time spent in moderate to heavy traffic based on the 24-h recall diary and supplemental questionnaire responses, though a greater amount of time was reported in the supplemental questionnaire portion of the survey. This could arise if respondents failed to recall some travel activities in the diary portion, but included the associated time in traffic with the supplemental questionnaire. Alternatively, respondents may overestimate time in traffic when not prompted by a sequential recall dairy. Similarly, a dietary survey study found greater reported intake of fruits and vegetables when assessed using questionnaires compared to 24-h dietary recall interviews (Eaton et al. 2013).

Confirmation of questionnaire responses regarding living near a roadway with moderate to heavy traffic was provided by LUR-based NO_2_ concentration estimates; mean NO_2_ exposure was greater for those who reported living within one block of a roadway with moderate to heavy traffic than those who did not and was similarly associated with household income. There are several potential limitations in the use of the national model developed by Hystad et al. (2011). First, the model was developed to predict mean annual concentrations from 2006. However, while the regional background values were based on 2006 monitoring data, the final estimates produced by the model were adjusted to account for local variation based on road network information, which did not vary significantly over time. Assignment of ambient concentrations using postal codes may introduce additional error due to the differences in the postal code size between different geographic areas. This source of error is likely minor in the reported analyses, because they were limited to urban areas, where postal codes typically reflect a single city block. Despite their potential limitations, these NO_2_ estimates have been applied widely in previous studies to elucidate associations between air quality and health (e.g., Ashley-Martin et al. 2016; Stieb et al. 2016; Chen et al. 2017b).

Response rates were low (Matz et al. 2014), in keeping with a downward trend for telephone surveys which has steepened in recent years (Pew Research Center 2012). Reduced response rates may introduce bias if respondents differ from non-respondents for variable(s) of interest. However, proper weighting and adjustment for non-response can allow for generalization of survey results to the entire target population despite low response rates (Pew Research Center 2012).

## Conclusions

Overall, the CHAPS 2 study provides quantitative estimates of time spent in traffic-influenced microenvironments by urban Canadians, both in vehicle and when engaged in active transportation. Previous studies have reported that total daily personal exposure was disproportionately impacted by time spent in transportation microenvironments. In this population representative study, we found that the proportion of daily time spent in transportation microenvironments was consistent with the previous exposure studies, suggesting that traffic emissions may be a major contributor to air pollution exposure for Canadians living in urban centers.

## Electronic supplementary material

ESM 1 (DOCX 34 kb)
