# Supplementary material for: Evaluation of daily time spent in transportation and traffic-influenced microenvironments by urban Canadians
Source: Air Qual Atmos Health. 2017 Nov 30;11(2):209–20. doi: 10.1007/s11869-017-0532-6 (PMC5847121; doi:10.1007/s11869-017-0532-6)
Supplement: Supplementary file 1 — (DOCX 34 kb) [file 11869_2017_532_MOESM1_ESM.docx]

Article: Evaluation of daily time spent in transportation and traffic-influenced microenvironments by urban Canadians

Journal: Air Quality, Atmosphere & Health

Authors: Carlyn J. Matz PhD*, David M. Stieb MD, Marika Egyed MSc, Orly Brion MSc, Markey Johnson PhD

*Corresponding author:

C. Matz (carlyn.matz@hc-sc.gc.ca)

Tel: 1-613-957-1882

Fax: 1-613-948-8482

Mail: Air Health Effects Assessment Division, Health Canada, 269 Laurier Ave W, PL 4903C, Ottawa, ON, K1A 0K9

**Supplemental Material**

**A) CHAPS 2 questions considered in analysis**

**Traffic question in 24-hr recall diary**

When a reported location is a vehicle (car, truck, van, motorcycle, bus, train, subway, rapid transit), active transportation (walking, running, bike, skateboard, rollerblade), waiting at a bus/train/ride stop, or being in a stroller/carried by an adult, a supplemental question is asked:

QT

Was this activity conducted on or near a roadway with moderate to heavy traffic?

(Surveyor Note: A roadway with "moderate to heavy traffic" is one that has a substantial amount of traffic for several hours of the day, such as a main thoroughfare, a busy boulevard, or a highway. It does not include quiet residential streets or roads with occasional traffic.)

[1] NO

[2] YES *[go to QTA]*

[88] DK

QTA

About how much of this activity was conducted on or near a roadway with moderate to heavy traffic?

[0] LESS THAN 1 MINUTE

[1-120] RECORD THE NUMBER OF MINUTES

[180] 3 HOURS

[240] 4 HOURS

[300] 5 HOURS

[360] 6 HOURS

[420] 7 HOURS

[480] 8 HOURS

[540] 9 HOURS

[600] 10 HOURS

[660] 11 HOURS

[720] 12 HOURS

[800] MORE THAN 12 HOURS

[88] DK

**Questions from supplemental questionnaire**

Traffic Block

TRAFFIC1

Thinking now about [fill name] time spent outdoors yesterday, were there any times when [fill name] [fill were] in a car, van, truck or bus in moderate to heavy traffic?

[1] NO *[go to Traffic2]*

[2] YES

[88] DK *[go to Traffic2]*

TRAFFIC1A

For how long altogether?

[1] LESS THAN 1 MINUTE

[2] RECORD THE NUMBER OF MINUTES

[180] 3 HOURS

[240] 4 HOURS

[300] 5 HOURS

[360] 6 HOURS

[420] 7 HOURS

[480] 8 HOURS

[540] 9 HOURS

[600] 10 HOURS

[660] 11 HOURS

[720] 12 HOURS

[800] MORE THAN 12 HOURS

[88] DK

TRAFFIC2

Were there any times yesterday when [fill name] [fill were] running, walking or standing alongside a road with moderate to heavy traffic?

[1] NO *[go to NEXT BLOCK]*

[2] YES

[88] DK *[go to NEXT BLOCK]*

TRAFFIC2A

For how long altogether?

[1] LESS THAN 1 MINUTE

[2] RECORD THE NUMBER OF MINUTES

[180] 3 HOURS

[240] 4 HOURS

[300] 5 HOURS

[360] 6 HOURS

[420] 7 HOURS

[480] 8 HOURS

[540] 9 HOURS

[600] 10 HOURS

[660] 11 HOURS

[720] 12 HOURS

[800] MORE THAN 12 HOURS

[88] DK

Parking Block

PARK1

Were there any times yesterday when [fill name] [fill were] in an enclosed parking garage or an indoor parking lot?

[1] NO *[go to Park2]*

[2] YES

[88] DK *[go to Park2]*

PARK1A

For how long altogether?

[1] LESS THAN 1 MINUTE

[2] RECORD THE NUMBER OF MINUTES

[180] 3 HOURS

[240] 4 HOURS

[300] 5 HOURS

[360] 6 HOURS

[420] 7 HOURS

[480] 8 HOURS

[540] 9 HOURS

[600] 10 HOURS

[660] 11 HOURS

[720] 12 HOURS

[800] MORE THAN 12 HOURS

[88] DK

PARK2

Were there any times yesterday when [fill name] [fill were] walking outside to a car in an open or outside parking area?

[1] NO *[go to NEXT BLOCK]*

[2] YES

[88] DK *[go to NEXT BLOCK]*

PARK2A

For how long altogether?

[1] LESS THAN 1 MINUTE

[2] RECORD THE NUMBER OF MINUTES

[180] 3 HOURS

[240] 4 HOURS

[300] 5 HOURS

[360] 6 HOURS

[420] 7 HOURS

[480] 8 HOURS

[540] 9 HOURS

[600] 10 HOURS

[660] 11 HOURS

[720] 12 HOURS

[800] MORE THAN 12 HOURS

[88] DK

Gas Station Block

GSTAT1

Did [fill name] spend ANY time yesterday at a gas station or auto repair shop?

[1] NO *[go to NEXT BLOCK]*

[2] YES

[88] DK *[go to NEXT BLOCK]*

GSTAT2

About how long altogether yesterday did [fill name] spend in those places?

[1] LESS THAN 1 MINUTE

[2] RECORD THE NUMBER OF MINUTES

[180] 3 HOURS

[240] 4 HOURS

[300] 5 HOURS

[360] 6 HOURS

[420] 7 HOURS

[480] 8 HOURS

[540] 9 HOURS

[600] 10 HOURS

[660] 11 HOURS

[720] 12 HOURS

[800] MORE THAN 12 HOURS

[88] DK

GSTAT3

Did you pump any gasoline yesterday at a gas station?

[1] NO

[2] YES

[88] DK

GSTAT4

[fill were] [fill name] in a car when another person was pumping gasoline?

[1] NO

[2] YES

[88] DK

Proximity to Roadway

BLOCK1

Is your home located on or within 1 block of a roadway with moderate to heavy traffic?

[1] NO

[2] YES

[88] DK

BLOCK1A (asked of respondents <18 years)

Is the school/daycare [fill name-child] attends located on or within 1 block of a roadway with moderate to heavy traffic?

[1] NO

[2] Child does not attend school or daycare

[3] YES

[88] DK

**B) Supplemental results**

Table S.1. Recall diary: Doer^a^ mean daily time spent in vehicle or vehicle-influenced microenvironments

| **Microenvironment** | **Weighted % of target population (N)** | **Mean daily time (min)** | **95% Confidence Interval (min)** | **95^th^%ile (min)** |
| --- | --- | --- | --- | --- |
| car | 59.5% (2116) | 77.3 | 71.6-83.1 | 208.1 |
| truck (pick up or van) | 9.0% (314) | 94.9^b^ | 51.6-138.2 | 383.5 |
| truck (not pick up or van) | 3.0% (113) | 129.6^b^ | 80.8-178.4 | 319.2 |
| motorcycle/moped/scooter | Data suppressed^c^ | | | |
| bus | 12.2% (347) | 66.3 | 51.2-81.5 | 173.8 |
| walking | 37.2% (1167) | 36.2 | 32.7-78.5 | 119.3 |
| bike/skateboard/rollerblade | 2.5% (63) | 59.2 | 40.0-78.5 | 188.0 |
| stroller/carried by adult | 0.4% (27) | 48.9^b^ | 27.1-70.7 | 107.7 |
| train/subway/rapid transit | 8.3% (163) | 70.1 | 59.1-81.2 | 123.1 |
| waiting at bus/train/ride stop | 2.9% (99) | 24.5 | 18.6-30.4 | 58.6 |
| running/jogging | 0.3% (13) | 38.2^b^ | 24.6-51.7 | 58.3 |
| Neighbourhood/sidewalk/street | 11.7% (439) | 54.7 | 46.8-62.6 | 134.9 |
| parking lot/open parking | 2.4% (118) | 28.3^b^ | 16.4-40.2 | 92.8 |
| service/gas station | 2.1% (87) | 10.7 | 7.8-13.6 | 20.9 |
| construction site | 0.8% (19) | 423.0^b^ | 263.5-582.4 | 633.1 |
| auto repair shop/gas station | Data suppressed^c^ | | | |
| enclosed parking structure | Data suppressed^c^ | | | |
| residential garage | 2.2% (96) | 46.4^b^ | 30.5-62.4 | 198.3 |

Note: ^a^ Those who reported spending time in the microenvironment

^b^ High sampling variability based on Statistics Canada guidelines for household surveys, interpret with caution

^c^ Due to very high sampling variability based on Statistics Canada guidelines for household surveys, data are suppressed

Statistics Canada. Guide to the Labour Force Survey. 2014. Catalogue no. 71-543-G.

Table S.2. Questionnaire: Time spent in a car, van, truck or bus in moderate to heavy traffic

| **Age Group** | **Survey Group** | **Weighted % of target population (N)** | **Mean time (95% CI) (min)** |
| --- | --- | --- | --- |
| 0-4 years | All respondents | 100% (334) | 18.4 (10.8-26.0) |
|  | Doers^a^ | 43.8% (165) | 42.1 (29.1-55.1) |
| 5-18 years | All respondents | 100% (522) | 25.2 (19.1-31.4) |
|  | Doers | 53.9% (308) | 47.0 (37.2-56.7) |
| 19-64 years | All respondents | 100% (1942) | 45.3 (35.7-55.0) |
|  | Doers | 63.8% (1294) | 71.3 (57.1-85.4) |
| 65+ years | All respondents | 100% (716) | 27.4 (23.0-31.7) |
|  | Doers | 51.9% (400) | 52.9 (46.3-59.4) |

Note: ^a^ Those who reported spending time in a car, van, truck or bus in moderate to heavy traffic

Table S.3. Questionnaire: Time spent running, walking, or standing along a roadside in moderate to heavy traffic

| **Age Group** | **Survey Group** | **Weighted % of target population (N)** | **Mean time (95% CI) (min)** |
| --- | --- | --- | --- |
| 0-4 years | All respondents | 100% (333) | 12.8^b^ (61.-19.5) |
|  | Doers^a^ | 27.5% (85) | 46.5^b^ (26.7-66.3) |
| 5-18 years | All respondents | 100% (520) | 11.2^b^ (7.3-15.0) |
|  | Doers | 32.9% (151) | 34.3 (24.1-44.4) |
| 19-64 years | All respondents | 100% (1936) | 14.4 (12.2-16.6) |
|  | Doers | 42.6% (752) | 33.9 (29.7-38.0) |
| 65+ years | All respondents | 100% (715) | 12.6^2^ (7.9-17.4) |
|  | Doers | 30.8% (200) | 41.2^2^ (27.1-55.3) |

Note: ^a^ Those who reported spending time running, walking, or standing along a roadside in moderate to heavy traffic

^b^ High sampling variability based on Statistics Canada guidelines (Statistics Canada 2014), interpret with caution

Table S.4. Questionnaire: Total combined time spent in a car, van, truck or bus in moderate to heavy traffic OR running, walking, or standing along a roadside in moderate to heavy traffic

| **Age Group** | **Survey Group** | **Weighted % of target population (N)** | **Mean time (95% CI) (min)** |
| --- | --- | --- | --- |
| 0-4 years | All respondents | 100% (334) | 31.3 (21.6-40.9) |
|  | Doers^a^ | 56.4% (189) | 55.2 (41.4-68.9) |
| 5-18 years | All respondents | 100% (522) | 36.4 (28.6-44.3) |
|  | Doers | 64.7% (352) | 56.3 (45.7-66.9) |
| 19-64 years | All respondents | 100% (1939) | 59.9 (49.4-70.5) |
|  | Doers | 75.9% (1484) | 79.0 (65.7-92.3) |
| 65+ years | All respondents | 100% (714) | 39.9 (33.4-46.5) |
|  | Doers | 62.9% (470) | 63.9 (54.9-72.9) |

Note: ^a^ Those who reported spending time in a car, van, truck or bus or spending time running, walking, or standing along a roadside in moderate to heavy traffic

Table S.5. Correlation between reported times in traffic: 24-h recall diary versus supplemental questionnaire

| **Group** | **Time in moderate to heavy traffic when in a vehicle** | **Total daily time in moderate to heavy traffic** |
| --- | --- | --- |
| All respondents | 0.7934 | 0.7790 |
| 0-4 years | 0.7742 | 0.7512 |
| 5-18 years | 0.8304 | 0.8111 |
| 19-64 years | 0.7880 | 0.7705 |
| 65+ years | 0.7680 | 0.7642 |
| Income ≤ LICO | 0.7743 | 0.7778 |
| Income > LICO | 0.7993 | 0.7822 |
| Employed full-time | 0.7951 | 0.7922 |
| Employed part-time | 0.7820 | 0.7695 |
| Not employed | 0.7676 | 0.7399 |
| Less than secondary education^a^ | 0.7083 | 0.7580 |
| Completed secondary education | 0.7852 | 0.7610 |
| Completed university | 0.7926 | 0.7819 |

Note: ^a^ Educational attainment was only ascertained for respondents ≥ 18 yrs

Table S.6. Mean difference between reported times in moderate to heavy traffic: 24-h recall diary versus supplemental questionnaire

| **Group** | **Time in moderate to heavy traffic when in a vehicle (min)**  **(Diary – Questionnaire)** | | | **Total daily time in moderate to heavy traffic (min)**  **(Diary – Questionnaire)** | | |
| --- | --- | --- | --- | --- | --- | --- |
|  | **N** | **Difference** | **95% Confidence Interval** | **N** | **Difference** | **95% Confidence Interval** |
| All respondents | 2518 | -9.1* | -18.3 – 0.0 | 2850 | -15.0* | -23.3 – (-6.8) |
| 0-4 years | 211 | 0.5 | -3.3 – 4.3 | 237 | -10.0* | -20.3 – 0.3 |
| 5-18 years | 374 | -5.4* | -9.3 – (-1.5) | 434 | -11.0* | -16.4 – (-5.6) |
| 19-64 years | 1468 | -12.0* | -25.3 – 1.3 | 1644 | -17.5* | -29.6 – (-5.4) |
| 65+ years | 465 | -0.9 | -5.6 – 3.8 | 535 | -8.1* | -13.1 – (-3.0) |
| Income ≤ LICO | 357 | -31.9 | -87.0 – 23.3 | 431 | -34.3 | -82.0 – 13.4 |
| Income > LICO | 1615 | -4.4* | -6.9 – (-2.0) | 1797 | -10.1* | -13.0 – (-7.4) |
| Employed full-time | 939 | -16.6 | -38.3 – 5.2 | 1031 | -21.9* | -41.8 – (-2.0) |
| Employed part-time | 256 | -5.3 | -12.2 – 1.6 | 287 | -7.8* | -14.4 – (-1.2) |
| Not employed | 722 | -2.9 | -6.5 – 4.1 | 842 | -11.5* | -16.9 – (-6.1) |
| Less than secondary education^a^ | 127 | 1.1 | -10.9 – 13.0 | 148 | -8.2 | -19.3 – 5.9 |
| Completed secondary education | 947 | -19.9 | -44.0 – 4.3 | 1059 | -24.7* | -46.6 – (-2.8) |
| Completed university | 822 | -2.6 | -5.7 – 0.5 | 933 | -8.8* | -12.9 – (-4.8) |

Note: ^a^ Educational attainment was only ascertained for respondents ≥ 18 yrs

* Statistically significant at p < 0.05
